# Cellular stress response, sirtuins and UCP proteins in Alzheimer disease: role of vitagenes

**DOI:** 10.1186/1742-4933-10-41

**Published:** 2013-10-17

**Authors:** Carolin Cornelius, Angela Trovato Salinaro, Maria Scuto, Vincenzo Fronte, Maria Teresa Cambria, Manuela Pennisi, Rita Bella, Pietro Milone, Antonio Graziano, Rosalia Crupi, Salvatore Cuzzocrea, Giovanni Pennisi, Vittorio Calabrese

**Affiliations:** 1 Department of Biomedical Sciences, University of Catania, Catania, Italy; 2 Department of Neuroscience, University of Catania, Catania, Italy; 3 Department of Medicine and Surgery, University of Catania, Catania, Italy; 4 Department of Clinical and Experimental Medicine and Pharmacology, School of Medicine, Messina, Italy

## Abstract

Alzheimer’s Disease (AD) is a neurodegenerative disorder affecting up to one third of individuals reaching the age of 80. Different integrated responses exist in the brain to detect oxidative stress which is controlled by several genes termed *Vitagenes*. Vitagenes encode for cytoprotective heat shock proteins (Hsp), as well as thioredoxin, sirtuins and uncouple proteins (UCPs). In the present study we evaluate stress response mechanisms in plasma and lymphocytes of AD patients, as compared to controls, in order to provide evidence of an imbalance of oxidant/antioxidant mechanisms and oxidative damage in AD patients and the possible protective role of vitagenes.

We found that the levels of Sirt-1 and Sirt-2 in AD lymphocytes were significantly higher than in control subjects. Interestingly, analysis of plasma showed in AD patients increased expression of Trx, a finding associated with reduced expression of UCP1, as compared to control group.

This finding can open up new neuroprotective strategies, as molecules inducing this defense mechanisms can represent a therapeutic target to minimize the deleterious consequences associated to oxidative stress, such as in brain aging and neurodegenerative disorders.

## Introduction

Alzheimer’s disease (AD) is a progressive neurodegenerative disorder and represents the most common cause of dementia in the elderly, accounting for 50-60% of all cases in Western world [1,2]. The prevalence rates for AD rise exponentially with age, increasing markedly after 65 years. AD is characterized by cognitive decline beginning usually with impairment of episodic memory, involving progressively all cognitive functions in the late stage [3]. Although some cases are familial, sporadic AD is more common, affecting more than 15 million people worldwide [4].

The pathological hallmarks of AD are amyloid plaques, containing amyloid-β peptide, derived from the transmebrane amyloid precursor protein, and neurofibrillary tangles, composed of hyperphosforylated tau protein, in the medial temporal lobe structures and cortical areas of the brain together with neuronal death and synapses loss [5,6]. Many approaches have been undertaken to understand AD, including Aβ aggregation, but the heterogeneity of the etiologic factors makes it difficult to define the clinically most important factors determining the onset and progression of the disease [7]. Accumulation of Aβ characterizes AD as a protein misfolding disease, suggesting a pathogenic role for oxidative stress in protein clearance defect by the ubiquitin-proteasome system [8,9]. In particular, misfolded Aβ is considered to be the key mediator of cellular oxidative stress in AD [10], and different evidences exist which indicate that oxidative stress is central to neurodegeneration in AD [11,12]. Consistently, increasing evidence indicates that factors such as oxidative stress and disturbed protein metabolism and their interaction in a vicious cycle are central to AD pathogenesis [13].

It is well known that living cells are continually challenged by conditions which cause acute or chronic stress. To adapt to environmental changes and survive different types of injuries, eukaryotic cells have evolved networks of different responses which detect and control diverse forms of stress [14]. One of these responses, known as the heat shock response, has attracted a great interest as a universal fundamental mechanism necessary for cell survival under a wide variety of toxic conditions [15-17]. Consistent with this, integrated survival responses exist in the brain, which are under control of redox regulated genes, called vitagenes, including heat shock proteins (Hsps), Sirtuins and Thioredoxin, that actively operate in detecting and controlling diverse forms of stress and neuronal injuries [17-19].

Sirtuins are a family of histone deacetylases that, in humans, includes at least seven members (silent information regulator two: SIRT 1-7) that exhibit different cellular and subcellular localizations and substrate specificities [20]. The best studied sirtuin is SIRT-1, an NAD + dependent enzyme that deacetylates several different protein substrates involved in the regulation of cellular energy metabolism and redox state, thereby influencing cell survival and plasticity [21-24]. Thioredoxin (Trx), is a major redox control system, consisting of a 12 kD a redox active protein Trx, and a homodimeric seleno-protein called thioredoxin reductase (TrxR1). TrxR1 is a flavoprotein that catalyzes the NADPH-dependent reduction of oxidized thioredoxin protein. It is usually located in the cytosol, but it translocates into the nucleus in response to various stimuli associated with oxidative stress. Trx, thus, plays a central role in protecting against oxidative stress [25,26].

Uncoupling proteins (UCPs) are members of the super family of anion carrier proteins located in the inner membrane of mitochondria. These proteins have several hypothesized functions including thermogenesis in certain tissues, protection from reactive oxygen species (ROS), neuroprotection and export of fatty acids. UCPs influence the production of mitochondrial reactive oxygen species. In general, the available data indicate that UCP activity results in decreased superoxide and hydrogen peroxide production [27,28]. In view of our previous finding demonstrating that in the brain and in peripheral blood significant changes in thiol status are associated with increased content of both protein and lipid oxidation markers, in the present study we measured the expression levels of stress responsive proteins such as sirtuin, thioredoxin and UCP protein in the blood of AD patients as compared to age-matched normal subjects to understand the potential role of these protective mechanism in the pathogenesis of AD pathology.

## Materials and methods

### Patients

The study was conducted according to guidelines of local Ethics Committee, and informed consent was obtained from all patients. Thirty patients (13 men and 17 women), with an age range of 69-81 years were enrolled in the study. All patients had progressive cognitive and memory impairment for at least 12 months and were diagnosed as suffering of probable AD, according to the criteria of the National Institute of Neurological and communicative Disorder and Stroke Alzheimer Disease and Related Disorder Association (NINCDS-ADRADA) (McKhann G, Drachman D, Folstein M, Katzman R, Price D, et al. Clinical diagnosis of Alzheimer’s disease: report of the NINCDS-ADRDA Work Group under the auspices of Department of Health and Human Services Task Force on Alzheimer’s Disease. Neurology. 1984;34:939-944.). The evaluation of the stage of dementia was assessed by the Mini Mental State Examination (MMSE) (“Mini-mental state”. A practical method for grading the cognitive state of patients for the clinician. Folstein MF, Folstein SE, Mc Hugh PR. J Psychiatr Res.1975 Nov;12 (3):189-98). Status of basic and instrumental activities of daily living (Activity of Daily Living, ADL, Instrumental Activity of Daily Living, IADL) was also assessed. None of our patients had a history of major psychiatric illness or other neurological disorders (i.e. Parkinson’s disease, stroke, dementia, multiple sclerosis, etc.), history of head trauma or epilepsy, acute or chronic medical illness, endocrinopathies or vitamin B deficiency affecting cognitive functions, alcohol or drug abuse, and conditions precluding MRI or CT execution. Three patients were classified as mild and 7 as moderate. All patients were under acetylcholinesterase inhibitor (AchE-I) medication. Computed tomography (CT) or magnetic resonance imaging (MRI) scan showed widespread cortical atrophy in most patients. In addition ten subjects (5 men and 5 women) with an age range of 60-79 years were studied as a control group. Controls showed no impairment in neuropsychological evaluation. Laboratory and neuroimaging tests were normal. The exclusion criteria of the control subjects were in line with those of patients. Clinicodemographic characteristics and neuropsychological test scores of patients and control subjects are shown in Table 1.

**Table 1:** Clinical and demographic data of AD patients and control subjects

	**Number of subjects**	**Age (mean ± SD)**	**Gender (F/M)**	**Disease duration (mean ± SD)**	**MMSE (mean ± SD)**	**ADL (mean ± SD)**	**IADL (mean ± SD)**
Patients	30	74.6 ± 4.28	17/13	2.7 ± 1.7	17.5 ± 3.8	4.9 ± 1.2	3.7 ± 2.9
Controls	10	69.3 ± 5.77	5/5		27.9 ± 2	5.6 ± 0.5	7.9 ± 0.3

MMSE: Mini Mental State Examination (normal values: >24/30).

ADL: Activity Daily Living (normal values: 6/6).

IADL: Instrumental Activity of Daily Living (normal values: 8/8).

### Sampling

Blood was collected from controls and patients by venipuncture from an antecubital vein into tubes containing EDTA as an anticoagulant. Immediately after sampling, 1 ml the blood was centrifuged at 3000 × g for 10 min at 4°C to separate plasma from red blood cells and 4 mL were utilized for lymphocytes purification. Lymphocytes from peripheral blood were purified using the Ficoll Paque System following the procedure provided by the manufacturer (GE Healthcare, Piscataway, NJ, USA).

### Lymphocyte purification

Lymphocytes from peripheral blood were purified by using the Ficoll Paque System following the procedure provided by the manufacturer (GE Healthcare, Piscataway, NJ, USA).

### Western blot analysis

Trx, Sirt-1, Sirt-2 were evaluated by Western blot analyses. Plasma samples were ready to use, while the lymphocyte pellet was homogenized (0,1 M NaCl, 0,01 M Tris Cl pH 7,6, 0,001 M EDTA pH 8, 100 μg/ml PMSF) and centrifuged at 10,000 × *g* for 10 min and the supernatant was used for analysis after dosage of proteins.

Equal concentrations of protein extracted for each sample (40 μg) were separated on a polyacrylamide mini gels precasting 4-20% (cod NB10420 NuSept Ltd Australia) using a miniprotean apparatus (BIO-RAD). Before being loaded on the gel, samples were boiled for 3 minutes in sample buffer (containing 40 mM Tris–HCl pH 7.4, 2.5% SDS, 5% 2-mercaptoethanol, 5% glycerol, 0.025 mg/ml of bromophenol blue). The proteins were transferred onto nitrocellulose membrane (0.45 μΜ) (BIO-RAD Hercules, CA, USA) in transfer buffer containing (0.05% di SDS, 25 mM di Tris, 192 mM glycine and 20% v/v methanol) using a miniprotean apparatus (BIO-RAD).

The transfer of the proteins on the nitrocellulose membrane was confirmed by staining with Ponceau Red which was then removed by 3 washes in PBS (phosphate buffered saline) for 5 min. each. The membranes were then incubated for 1 hour at room temperature in 20 mM Tris pH 7.4, 150 mM NaCl and Tween 20 (TBS-T) containing 2% milk powder and incubated with appropriate primary antibodies, namely anti-Trx, anti Sirt-1, anti Sirt-2, anti UCP1 rabbit polyclonal antibody (Santa Cruz Biotech. Inc., Santa Cruz, CA, USA), overnight at 4°C in TBS-T.

The same membrane was incubated with a goat polyclonal antibody anti-beta-actin (SC 1615 Santa Cruz Biotech. Inc., CA, USA, dilution 1:1000) to verify that the concentration of protein loaded in the gel was the same in each sample.

Excess unbound antibodies were removed by 3 washes are with TBS-T for 5 minutes. After incubation with primary antibody, the membranes were washed 3 times for 5 min. in TBS-T and then incubated for 1 h at room temperature with the secondary polyclonal antibody conjugated with horseradish peroxidase (dilution 1:500).

The membranes were then washed 3 times with TBS-T for 5 minutes. Finally, the membranes were incubated for 3 minutes with SuperSignal chemiluminiscence detection system kit (Cod 34080 Pierce Chemical Co, Rockford, USA) to display the specific protein bands for each antibody. The immunoreactive bands were quantified by capturing the luminescence signal emitted from the membranes with the Gel Logic 2200 PRO (Bioscience) and analyzed with Molecular Imaging software for the complete analysis of regions of interest for measuring expression ratios. The molecular weight of proteins analyzed was determined using a standard curve prepared with protein molecular weight.

### Determination of protein

Samples protein concentrations were determined by the bicinchoninic acid protein assay (Cod 23227 Pierce Protein Research Products, Rockford, IL 61101 U.S.A.) according to the method described in Smith et al. [29] and using bovine serum albumin as standard.

### Statistical analysis

All results are expressed as means ± S.E.M. Each experiment was performed, unless otherwise specified, in triplicate. Data were analyzed by one-way ANOVA, followed by inspection of all differences by Duncan’s new multiple-range test. Differences were considered significant at P < 0.05.

## Results

**Figure 1:**
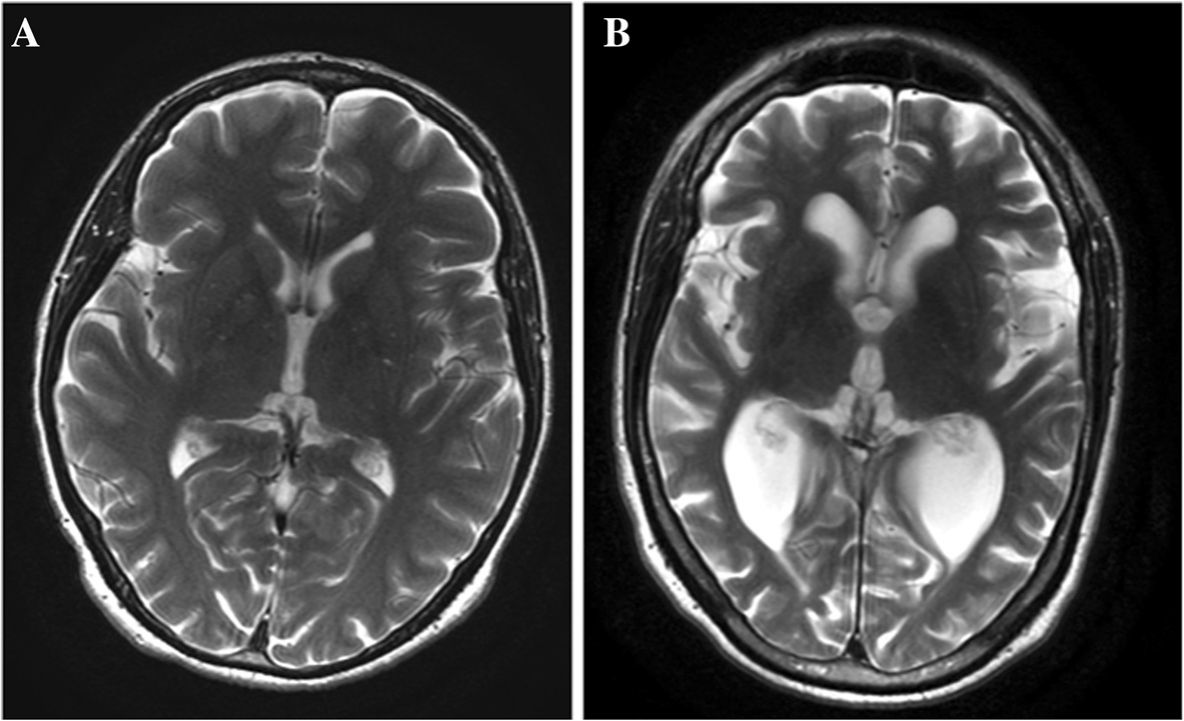
**Brain MRI.** Axial T2 images shows cerebral atrophy in patient with Alzheimer’s disease **(A)** and normal brain in control patient of same age **(B)**.

**Figure 2:**
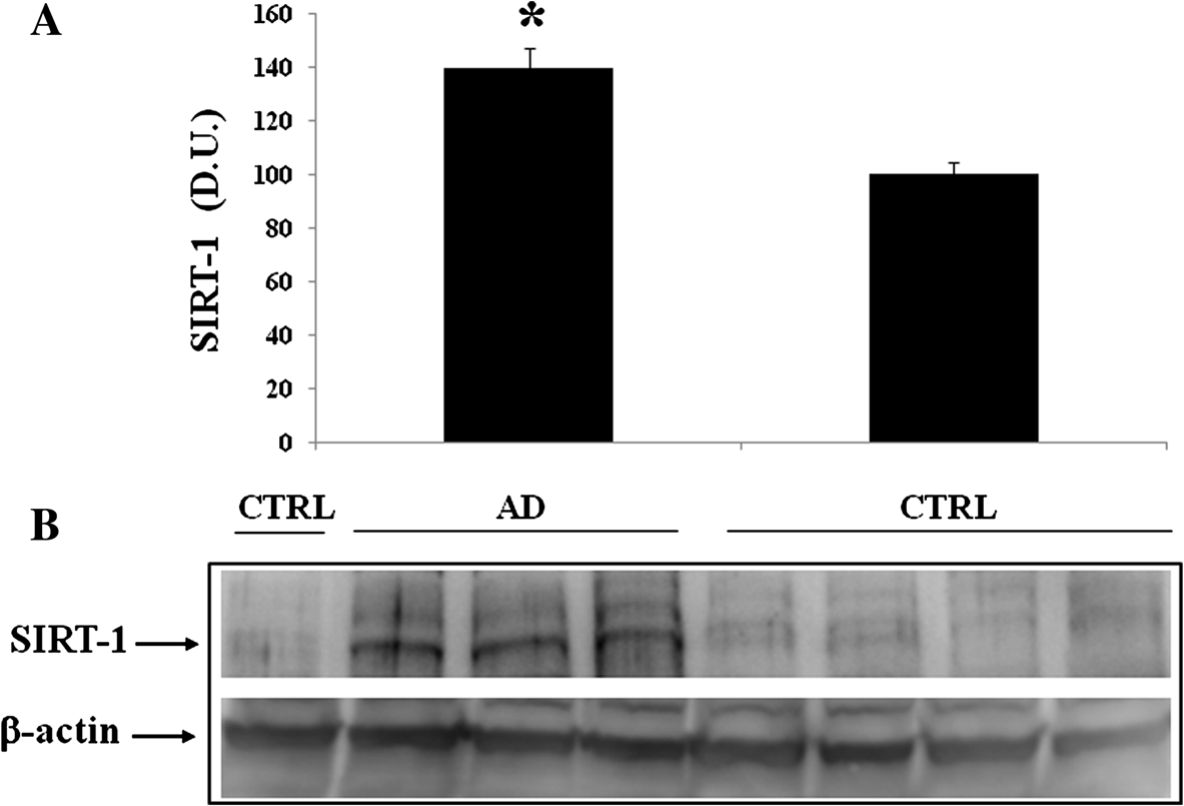
**Sirtuin-1 (Sirt-1) protein levels in lymphocytes of AD and control subjects.** Samples from control and AD patients were assayed for Sirt-1 expression by Western blot. **A)** Densitometric evaluation: the bar graph shows the values are expressed as mean standard error of mean of 3 independent analyses. *P* ≤ 0.05 vs control. **B)** A representative immunoblot is shown. β-actin has been used as loading control. D.U., densitometric units; AD, Alzheimer’s disease; CTRL, control.

**Figure 3:**
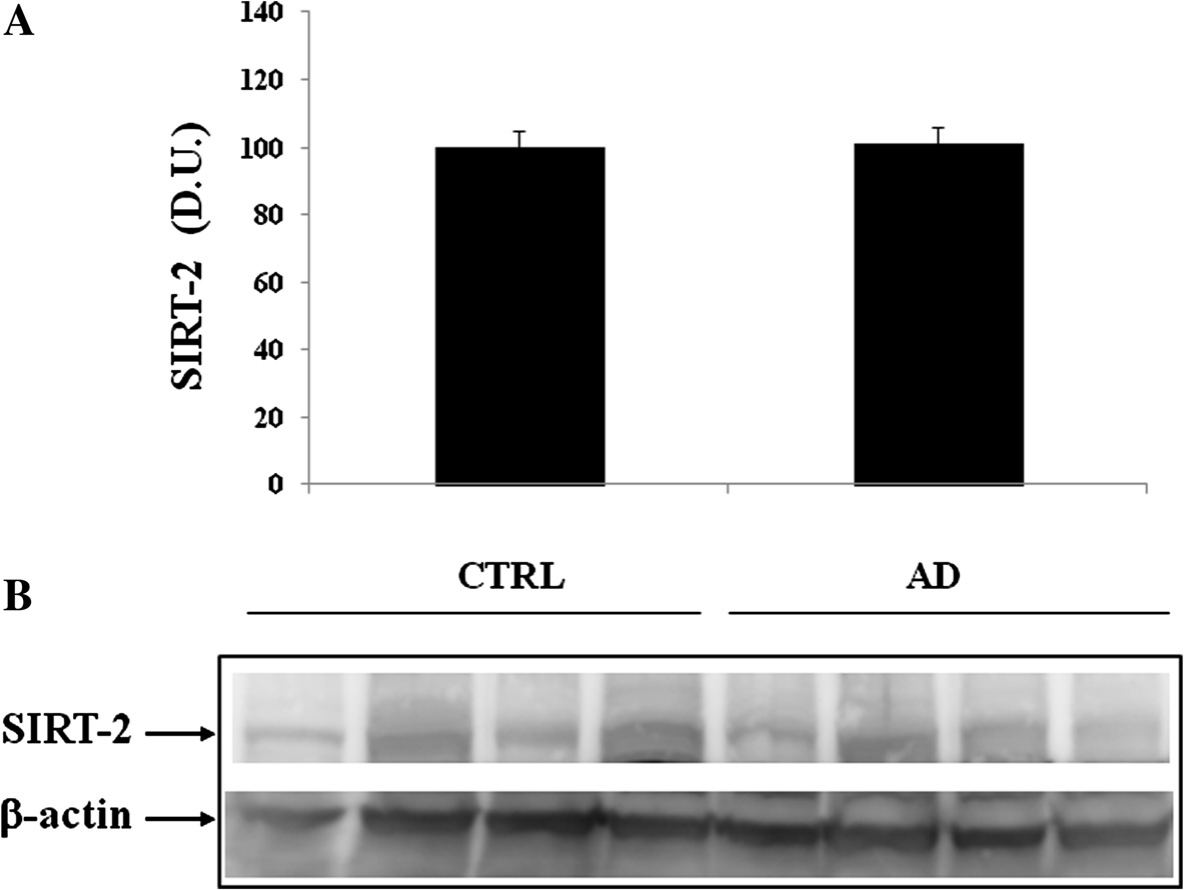
**Sirtuin-2 (Sirt-2) protein levels in lymphocytes of AD and control subjects.** Samples from control and AD patients were assayed for Sirt-2 expression by Western blot. **A)** Densitometric evaluation: the bar graph shows the values are expressed as mean standard error of mean of 3 independent analyses. **B)** A representative immunoblot is shown. β-actin has been used as loading control. D.U., densitometric units; AD, Alzheimer’s disease; CTRL, control.

**Figure 4:**
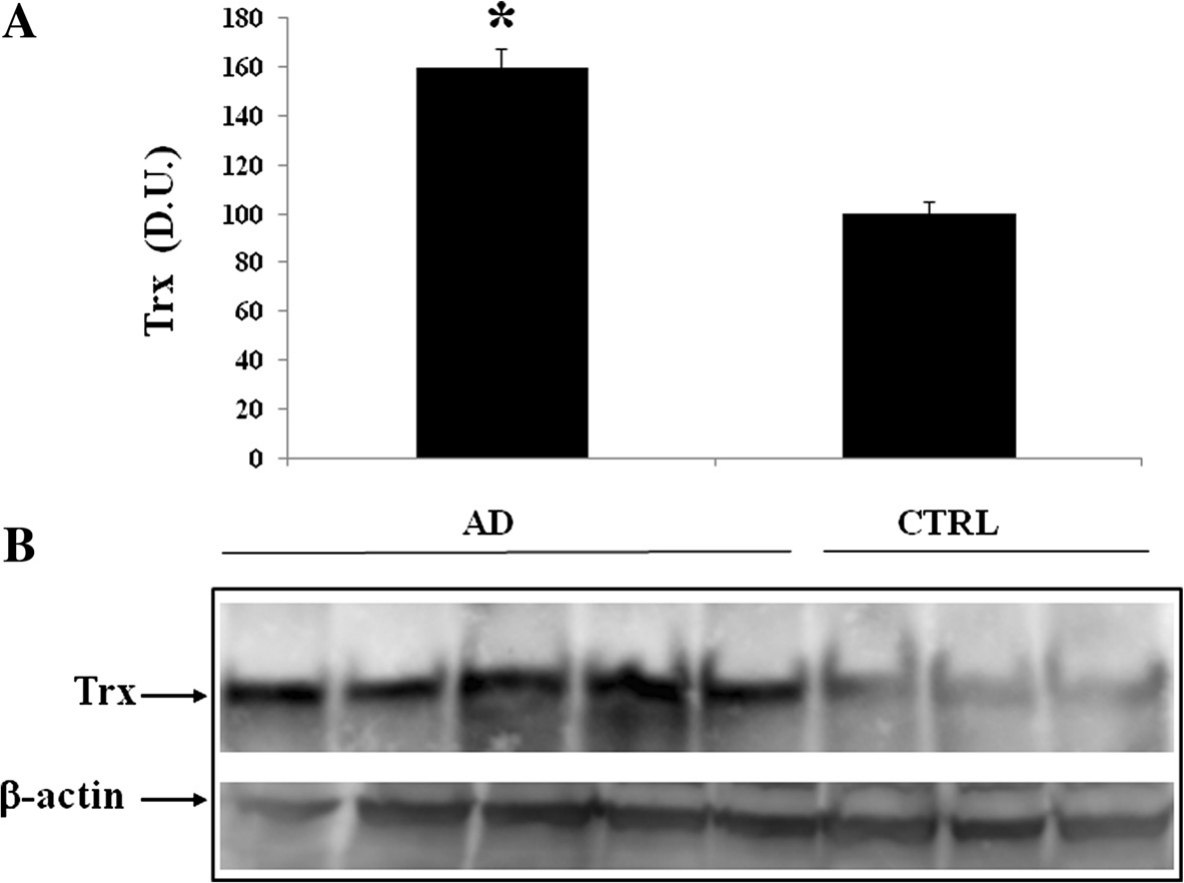
**Thioredoxin (Trx) protein levels in lymphocytes of AD and control subjects.** Samples from control and AD subjects were assayed for Trx expression by Western blot. **A)** Densitometric evaluation: the bar graph shows the values are expressed as mean standard error of mean of 3 independent analyses. *P* ≤ 0.05 vs control. **B)** A representative immunoblot is shown. β-actin has been used as loading control. D.U., densitometric units; AD, Alzheimer’s disease; CTRL, control.

**Figure 5:**
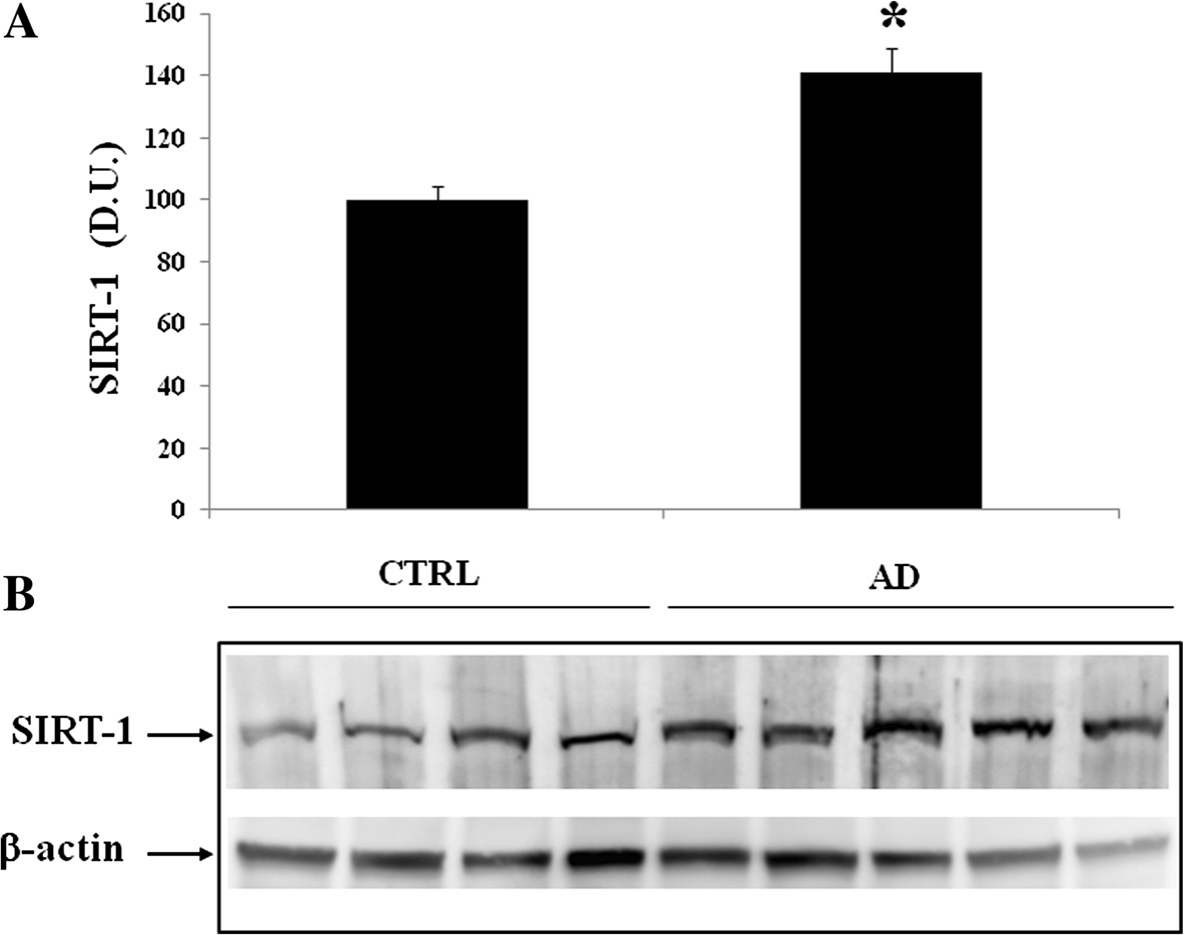
**Plasma levels of Sirtuin-1 (Sirt-1) in AD and control individuals.** Samples from control and AD subjects were assayed for Sirt-1 expression by Western blot. **A)** Densitometric evaluation: the bar graph shows the values are expressed as mean standard error of mean of 3 independent analyses. *P* ≤ 0.05 vs control. **B)** A representative immunoblot is shown. β-actin has been used as loading control. D.U., densitometric units; AD, Alzheimer’s disease; CTRL, control.

**Figure 6:**
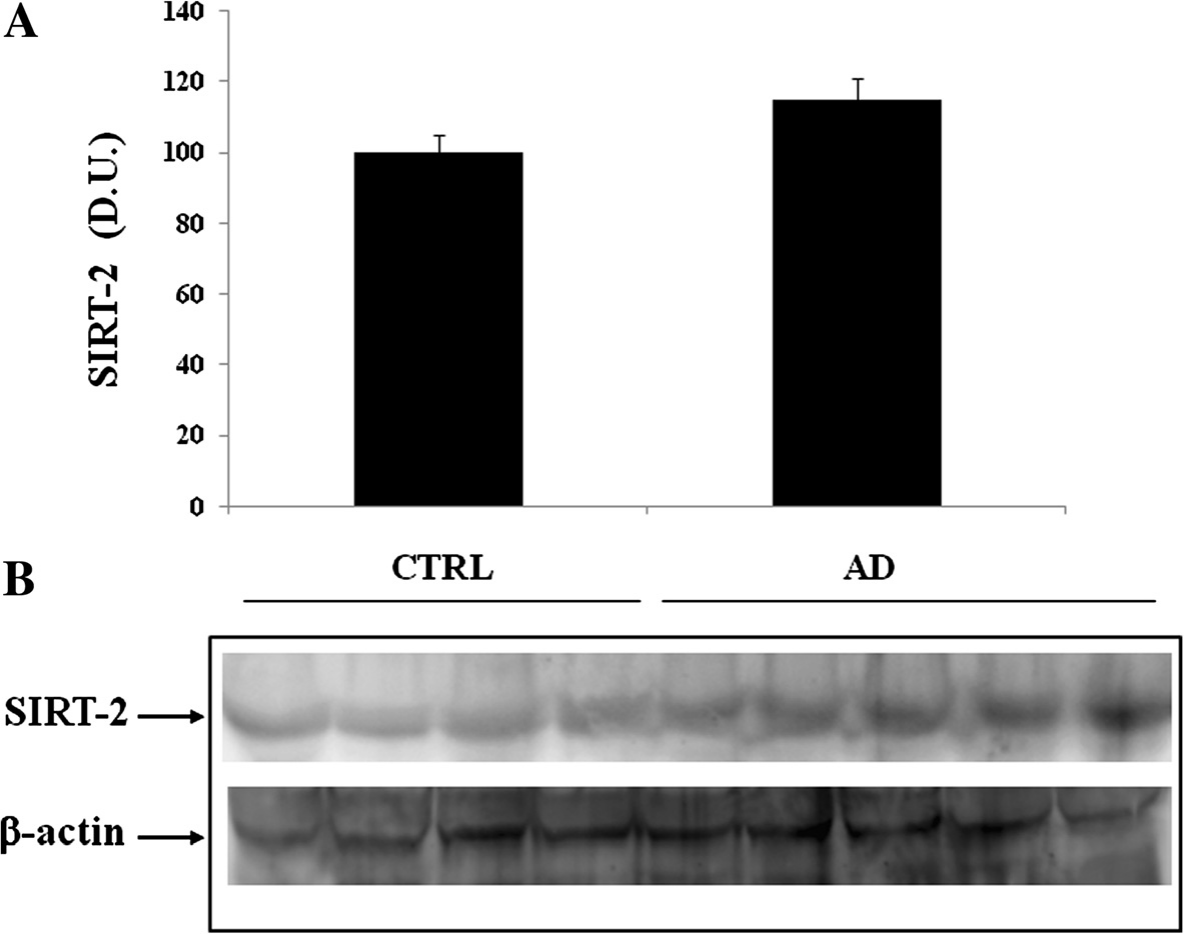
**Plasma levels of Sirtuin-2 (Sirt-2) in AD and control individuals.** Samples from control and AD subjects were assayed for Sirt-2 expression by Western blot. **A)** Densitometric evaluation: the bar graph shows the values are expressed as mean standard error of mean of 3 independent analyses. **B)** A representative immunoblot is shown. β-actin has been used as loading control. D.U., densitometric units; AD, Alzheimer’s disease; CTRL, control.

**Figure 7:**
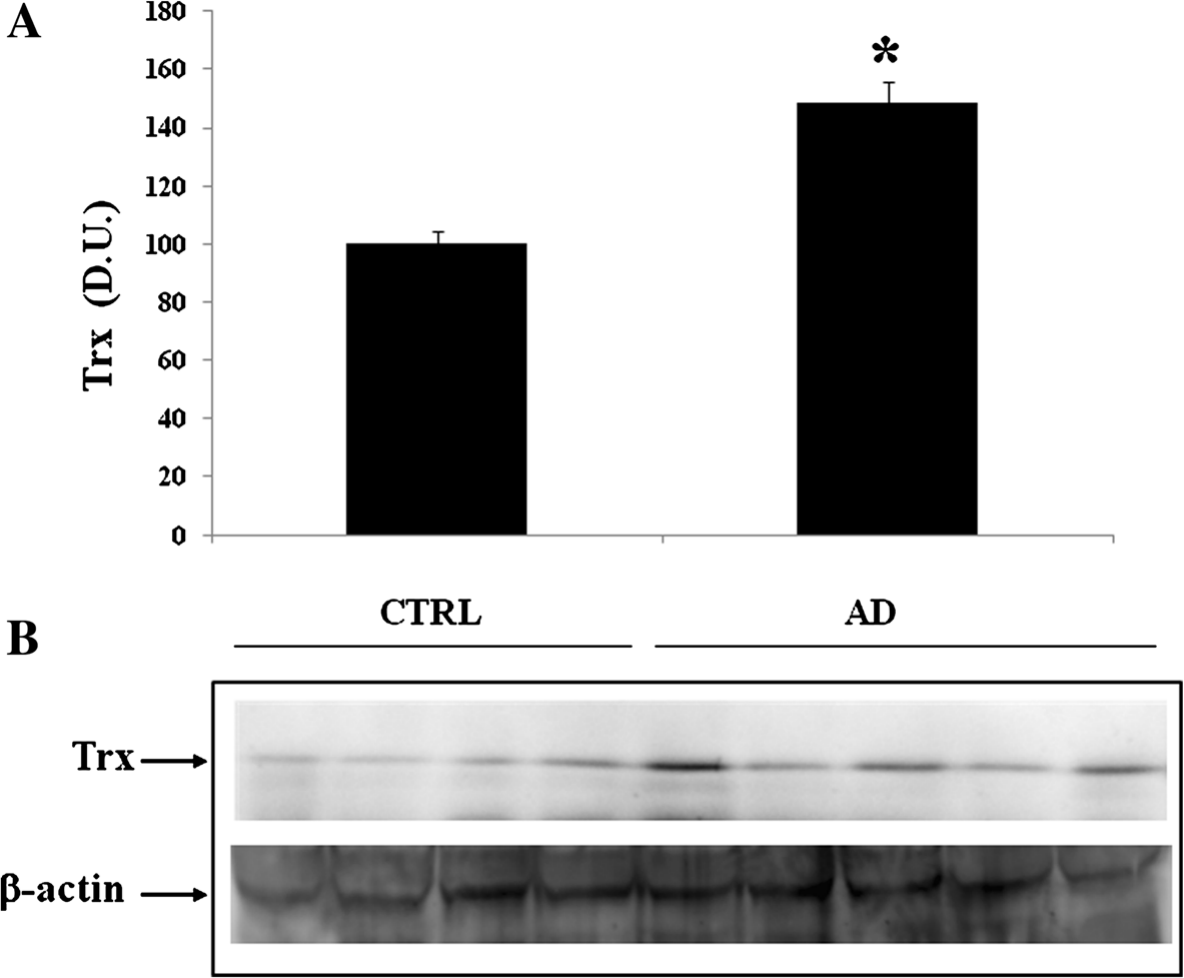
**Plasma levels of Thioredoxin (Trx) in AD and control individuals.** Samples from control and AD subjects were assayed for Trx expression by Western blot. **A)** Densitometric evaluation: the bar graph shows the values are expressed as mean standard error of mean of 3 independent analyses. *P* ≤ 0.05 vs control. **B)** A representative immunoblot is shown. β-actin has been used as loading control. D.U., densitometric units; AD, Alzheimer’s disease; CTRL, control.

**Figure 8:**
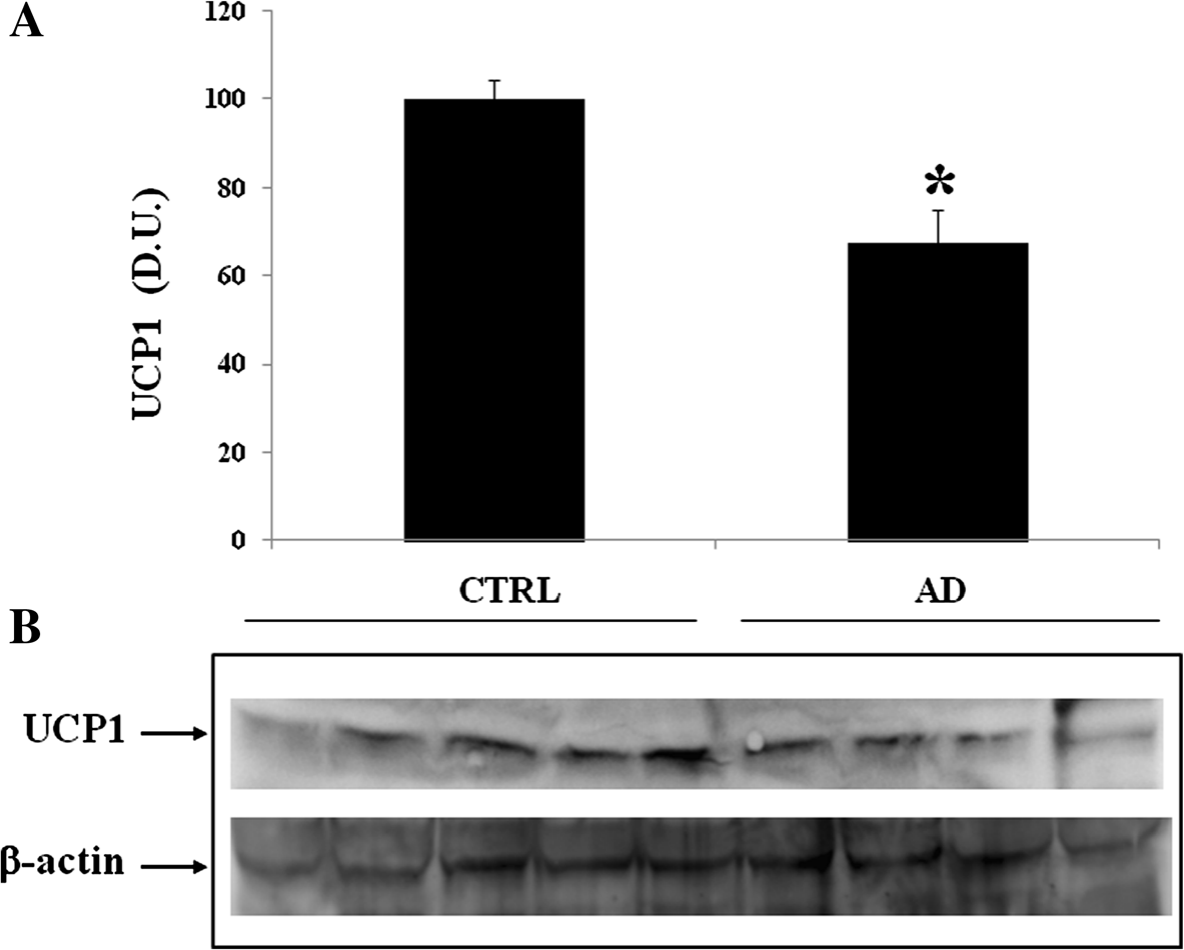
**Uncoupling proteins 1 (UCP1) levels in the plasma of AD and control individuals.** Samples from control and AD were assayed for UCP1 expression by Western blot. **A)** Densitometric evaluation: the bar graph shows the values are expressed as mean standard error of mean of 3 independent analyses. **B)** A representative immunoblot is shown. β-actin has been used as loading control. D.U., densitometric units; AD, Alzheimer’s disease; CTRL, control.

Alzheimer’s disease (AD) is the most common form of dementia and is characterized pathologically by senile plaques, neurofibrillary tangles and cerebral amyloid angiopathy [30-32]. Figure 1 reports brain MRI axial T2 image showing cerebral atrophy in patient with Alzheimer’s disease in comparison to a normal brain. Our laboratory previously demonstrated in the brain as well as in peripheral blood that oxidative and nitrosative stress occur in AD patients, compared to normal subjects [33] and that this can serve as a trigger for induction of the heat shock response [18,34,35]. Therefore, we evaluated the expression levels of Trx and Sirtuin in the plasma and lymphocytes in control and in AD patients. Western blot analysis of lymphocytes probed for Sirt-1 is reported in Figure 2. Sirt-1 expression is significantly increased in AD patients, compared to controls. In contrast to Sirt-1, expression levels of Sirt-2 measured in lymphocytes did not show a significant increase in AD patients compared to controls (Figure 3). As shown in Figure 4, analysis of lymphocytes in AD patients, compared to control group, revealed also an increase in thioredoxin protein expression. Consistently to the observed changes in AD lymphocytes, analysis of plasma in AD patients showed higher expression levels of Sirt-1 (Figure 5). Expression levels of Sirt-2 were also measured and results, reported in Figure 6, show an increase in AD patients, which however was not statistically significant, as compared to control group. As far as we are concerned, this is the first evidence demonstrating changes in SIRT-1 expression in AD, although at the moment we cannot exclude that this might not be a specific alteration of this progressive inflammatory neurodegenerative disease associated with oxidative stress which has emerged as a critical factor in AD. Interestingly, we investigated the expression of Trx and we found, in the plasma, higher levels of Trx protein in AD patients compared with the control group (Figure 7). Figure 8 shows a decreased expression of UCP1 protein in plasma of AD patients compared to controls. Analysis of lymphocytes in AD patients, compared to control group, did not allow to detect measurable levels of this protein (data not shown).

## Discussion

Alzheimer’s disease is a progressive disorder characterized usually by early memory loss, however affecting all intellectual functions in the late stage and leadind to complete dependence for basic functions of life. The pathological features of AD are a variable degree of cortical atrophy, in the frontal, parietal, and temporal lobes (Figure 1). The pathological lesions in AD include neurofibrillary tangles, neurite, plaques, the central core of which is amyloid-β peptide, derived from the transmembrane amyloid precursor protein (APP), amyloid angiopathy [7,13]. AD brain has been reported to be under oxidative stress that may play an important role in the pathogenesis and progression of AD [14,36,37]. Several lines of evidence support a fundamental role for free radical mediated event in the pathogenesis of the disease. Amyloid-β peptide [1,2,4-8,10,11,14-27,30,33], [34,36-51] has been shown to induce protein oxidation in both *in vitro* and *in vivo* studies [40-42,48]. As a result, amyloid-β peptide [1,2,4-8,10,11,14-27,30,33], [34,36-51] has been proposed to play a central role in the pathogenesis of AD [43]. We have previously shown that increased protein oxidation and lipid peroxidation are present in the brain from patients with mild cognitive impairment (MCI), as compared to aged-matched control brain [44,51]. Because many researchers consider MCI to be the transition zone between normal cognition and the dementia of early AD [45,46].

Cells have evolved different adaptive responses to manage oxidative stress which includes the recognition of cellular redox potential, reactive oxygen species and protein oxidation and responding with changes in gene expression [52,53]. Sirt-1 and Sirt-2 are stress induced proteins that have been implicated in defense mechanisms against agents that may induce oxidative injury, and its induction represents a common feature in a number of neurodegenerative diseases [54]. In addition, another protein, thioredoxin reductase (TrxR), is emerging as critical vitagene involved in brain stress tolerance. As such, it has been demonstrated that Trx plays an important role in protecting against oxidative stress and in regulating cell growth and cell death [38,55]. In the present study, the role of the vitagenes Sirt-1, Sirt-2 and Trx, was investigated in the peripheral blood of AD patients to gain further insight into the role of oxidant/antioxidant balance as critical factors operating in the pathogenesis of AD. We found that the levels of Sirt-1 and Sirt-2 in AD lymphocytes were significantly higher than in control patients a finding associated with increased expression of Trx, and a reduced expression of UCP1, as compared to control group. The increased expression of these proteins, however, appear to be consequence of a strong oxidant environment, which can be relevant to the pathogenesis of AD. Sirt-1, has received considerable attention, as it has been recently demonstrated that Sirt-1 induction could represent a protective system potentially active against brain oxidative injury [20,39,56]. Several studies suggest that the Sirt-1 gene is redox-regulated and its expression appears closely related to conditions of oxidative stress [49,57]. Another protein, in addition, thioredoxin reductase (Trx), is emerging as critical vitagene involved in brain stress tolerance. As such, it has been demonstrated that Trx plays an important role in protecting against oxidative stress and in regulating cell growth and cell death [50,55,58]. Furthermore, we found decreased levels of UCP expression in AD patients. Uncoupling proteins (UCPs) are members of the family of mitochondrial anion carrier proteins. The UCP1 is an integral membrane protein unique to brown adipose tissue mitochondria. UCP1 separates oxidative phosphorylation from ATP synthesis with energy dissipated as heat. UCP1 facilitates the transfer of anions from the inner to the outer mitochondrial membrane and the return transfer of protons from the outer to the inner mitochondrial membrane. UCP1 is activated in the brown fat cell by fatty acids and inhibited by nucleotides [47]. Mitochondrial uncoupling mediated by uncoupling protein 1 (UCP1) is classically associated with non-shivering thermogenesis by brown fat. UCP family proteins are also present in selected neurons. They can be activated by free radicals and free fatty acids, and their activity has a profound influence on neuronal function. By regulating mitochondrial biogenesis, calcium flux and local temperature, neuronal UCPs can directly influence neurotransmission, synaptic plasticity and neurodegenerative processes. In addition, by reducing free radical generation, UCP protein may serve a cytoprotective system. Our results demonstrate that AD is associated with increased oxidative stress, which could have an impact on mitochondrial bioenergetics affecting the function of neuronal mitochondrial complex IV and complex V [47]. In this context, simultaneous reductions in cytoprotective mechanisms, such as the UCP system, could allow oxidative injury to go unchecked or increase over time, thus representing an important factor sustaining the oxidative stress hypothesis of AD pathogenesis. Consistently, modulation of endogenous cellular defense mechanisms such as the vitagene network, including sirtuin, thioredoxin and UCP proteins may have the potential to broaden up new approaches to therapeutic interventions in diseases associated with tissue damage and cell death, such as in neurodegeneration. Our data, supporting a role for oxidative stress in the pathogenesis of AD, indicate that the stress responsive genes may represent an important target for novel cytoprotective strategies, as molecules inducing this defense mechanism, via nutritional and/or pharmacological approaches, can exploit the potential for antidegenerative therapeutic effects.

## Abbreviations

Aβ: Amyloid-β; AchE-I: Acetylcholinesterase inhibitor; AD: Alzheimer’s disease; APP: Amyloid precursor protein; CNS: Central nervous system; CR: Calorie restriction; CT: Computed tomography; CTRL: Control; DNPH: 2,4-dinitrophenylhydrazine; D.U: Densitometric units; Hsp: Heat shock proteins; IADL: Activity of daily living, ADL, Instrumental activity of daily living; MCI: Mild cognitive impairment; MMSE: Mini mental state examination; MRI: Magnetic resonance imaging; NINCDS-ADRADA: National Institute of Neurological and communicative Disorder and Stroke Alzheimer Disease and Related Disorder Association; NRF-2: Nuclear factor-erythroid 2-related factor 2; ROS: Reactive oxygen species; Sirt-1: Sirtuin-1; Sirt-2: Sirtuin-2; Trx: Thioredoxin; TrxR1: Thioredoxin reductase 1; UCP1: Uncoupling proteins.

## Competing interests

The authors declare that they have no competing interests.

## Authors’ contributions

CC contributed to the study design, to all experiments and to all sample analyses; AT, MS, VF, and MTC, contributed to the experiments and sample analyses; MP, RB, PM AG, RC, and SC contributed to the study design; GP coordinated the clinical managements of patients and the study design together with VC who leaded all experiment phases, and organization of the study design. All authors read and approved the final manuscript.
